# The Utility of Diffusion-Weighted MRI Lesions to Compare the Effects of Different Heparinization Schemes in Intracranial Aneurysms Treated by Endovascular Intervention

**DOI:** 10.3389/fneur.2020.609384

**Published:** 2020-12-10

**Authors:** Linfeng Zhang, Xiaobing Zhou, Yong Liu, Cong Ding, Yang Wang, Hongchao Yang

**Affiliations:** ^1^Department of Neurosurgery, The First Affiliated Hospital of Nanchang University, Nanchang University, Nanchang, China; ^2^School of Public Health, Nanchang University, Nanchang, China; ^3^Department of Neurosurgery, Beijing Chaoyang Hospital, Capital Medical University Beijing, Beijing, China

**Keywords:** intracranial aneurysm, heparinization, diffusion-weighted imaging, endovascular treatment, vascular disorders

## Abstract

**Objective:** Heparinization is applied to prevent ischemic complications in the endovascular treatment of intracranial aneurysms, but there is no unified heparinization scheme. Diffusion-weighted imaging (DWI) can be used to evaluate ischemia after endovascular therapy for intracranial aneurysms. The goal of this study is to apply DWI to evaluate the effects of different heparinization schemes on intracranial aneurysms treated with endovascular therapy.

**Methods:** We retrospectively reviewed 141 patients with 149 aneurysms treated with endovascular interventions from July 2019 to April 2020 at our center, including 96 aneurysms treated with local heparinization and 53 aneurysms treated with systemic heparinization. We collected the basic information of the patients, including age, sex, comorbidities, and aneurysm characteristics, and associated treatment data. New ischemic lesions detected by DWI were categorized belonging to four types. Multivariate logistic regression was used to compare the effects of different heparinization schemes on intracranial aneurysms treated with endovascular therapy.

**Results:** There were no significant differences in age, sex, hypertension, diabetes, and aneurysm size or location between the two groups. The incidence and distribution types of DWI abnormalities in the local heparinization groups and systemic heparinization groups were not significantly different (*P* > 0.05). There was a correlation between the laser engraving stent and postoperative DWI abnormalities (*P* < 0.003). Multivariate logistic regression analysis showed that the laser engraving stent was significantly correlated with postoperative DWI abnormalities (odds ratio, 4.71; 95% CI: 1.51–14.58; *P* = 0.007).

**Conclusion:** Compared with systemic heparinization, local heparinization does not increase the incidence of DWI abnormalities after endovascular treatment, and its application in this group of patients is safe and effective.

## Introduction

Endovascular treatment has become the main treatment method for intracranial aneurysms, but ischemic complications are the main risk of endovascular treatment (1). Diffusion-weighted imaging (DWI) is a sensitive method for detecting acute ischemic strokes. Studies have found that the incidence of ischemic lesions is between 10 and 77% after endovascular treatment of intracranial aneurysms, and ischemic lesions may be associated with cognitive decline, depression, and future stroke in patients (2–5). The causes of these associations of ischemic lesions include fragile plaques, thrombosis exfoliation in aneurysms, and thrombosis caused by the catheter (6). Previous studies have also shown that age, aneurysm size, and use of complex embolization techniques are risk factors for ischemic complications (7, 8).

Heparinization is an important method to prevent ischemic complications; however, there is no unified scheme for heparinization. The commonly used schemes are systemic heparinization and local heparinization. The goal of this study is to apply DWI to evaluate the effects of different heparinization schemes on intracranial aneurysms with endovascular treatment.

## Methods

We retrospectively collected patients who received endovascular treatment for intracranial aneurysm in our center from July 2019 to April 2020. The inclusion criteria were a saccular intracranial aneurysm (for ruptured aneurysms, the Hunt-Hess grade was 0–2) and MRI examination within 72 h after operation, while the exclusion criteria were dissecting aneurysm, blood blister-like aneurysm, aneurysms with moyamoya disease or arteriovenous malformation, previous use of antithrombotic drugs (including anticoagulants or antiplatelet aggregation drugs), or no MRI examination. The clinical data of the patients were collected, including age, sex, combined diseases (hypertension, diabetes, and hyperlipidemia), history of stroke, aneurysm characteristics, heparinization method, operation time, intraoperative hypotension, and endovascular technique. The aneurysms were classified according to location as anterior cerebral artery aneurysms (including anterior communicating aneurysm and A1 segment of anterior cerebral artery and distant arterial aneurysm), middle cerebral artery aneurysms, internal carotid artery aneurysms (including cavernous sinus segment aneurysm, clinoid process segment aneurysm, ophthalmic artery segment aneurysm, and communicating segment aneurysm) and posterior circulation aneurysms (including vertebral artery aneurysm, basilar artery aneurysm, post-erosuperior cerebellar artery aneurysm and posterior cerebral aneurysm). An intraoperative duration of more than 120 min was used as a marker of a long duration operation. The diagnostic criterion for intraoperative hypotension were systolic blood pressure <90 mmHg or a systolic blood pressure drop of more than 1/3 of baseline, except for anesthetic factors. This study was supported by our hospital, and all patients provided informed consent.

In the local heparinization group, no antiplatelet drugs were used before operation, and the catheter flushing solutions contained 2,000 U of heparin per 500 ml of fluid during the operation. For patients treated with coiling alone in the local heparinization group, no additional heparin or antiplatelet therapy was given during the perioperative period. For patients treated with stent-assisted embolization in the local heparinization group, tirofiban (10 μg/kg) was given during the operation and was continuously administered via a venous pump (0.15 μg/kg/min) 1 h later. A loading dose of aspirin (300 mg) and clopidogrel (300 mg) was given 6 h after the operation. Tirofiban and antiplatelet drugs should be used in combination and were overlapped at least 6 h before stopping antiplatelet drugs. The scheme of the systemic heparinized group was treated with intravenous injection according to the standard heparinization regimen (70 U/kg); then, 1,000 U heparin was administered every hour. For patients treated by stent-assisted embolization in the systemic heparinized group, aspirin (100 mg/days) and clopidogrel (75 mg/days) were administered for 3–7 days before the procedure. After the operation, daily clopidogrel (75 mg) was maintained for 1 month, and daily aspirin (100 mg) was maintained for 6 months. For patients treated with coiling alone in the systemic heparinized group, no antiplatelet therapy was given before or after the operation.

A laser engraving stent and dense network stent were used in the procedure. The laser engraving stent was either the Enterprise stent (Codman; USA), Solitaire stent (EV3; USA), or Neuro Form EZ stent (Boston Scientific; USA). The dense network stent either the LVIS stent (Microvention; USA), Pipeline stent (Medtronic; USA), or Tubridge stent (MircoPort; Shanghai).

All patients received an MRI examination with diffusion-weighted imaging (MRI-DWI), 1.5T or 3.0T, within 72 h after endovascular treatment. All MRIs were reviewed by two experienced neuroradiologists. Ischemic lesions were identified by DWI and were categorized as the following types: (A) unilateral 1~3 DWI points <10 mm; (B) unilateral >3 DWI points <10 mm or flaky DWI points >10 mm; (C) bilateral 1~3 DWI points <10 mm; and (D) bilateral >3 points <10 mm or flaky DWI points >10 mm.

### Statistical Analysis

The independent samples *T-*test was applied for comparison of continuous variables, and the chi-square test or Fisher's exact test was used for analysis of categorical variables. Logistic regression was used for multiple analysis to evaluate the relation between asymptomatic ischemic lesions and other factors after endovascular treatment. A *p* < 0.05 was considered significant. We used standard commercial software SPSS version 22 (IBM Corp., Armonk, NY, USA).for statistical analysis.

## Results

During the study period, a total of 255 patients received endovascular treatment for intracranial aneurysms at our center, and 141 patients with 149 aneurysms met the inclusion criteria. The mean age was 58.7 ± 10.3 years, and 102 (72.3%) patients were female. Endovascular operations were performed in 149 aneurysms (ruptured and unruptured aneurysms), including 41 anterior communicating artery aneurysms, 26 middle cerebral aneurysms, 73 internal carotid artery aneurysms, and nine posterior circulation aneurysms. Of all aneurysms, 72 aneurysms received coil embolization, and 77 aneurysms were treated by stent-assisted embolization, including 43 (55.8%) laser engraving stents (38 Enterprise stents, three Neuro Form EZ stents, and two Solitaire stents) and 34 (44.2%) dense network stents (31 LVIS stents, one Pipeline stent, and two Tubridge stents).

In total, 91 patients with 96 aneurysms were treated with endovascular embolization in the local heparinization group, and 50 patients with 53 aneurysms were treated with endovascular embolization in the systemic heparinization group. The demographic parameters were evenly distributed among the groups (female: 69.2 vs. 78.0%, *p* > 0.05; age: 59.74 ± 10.7 years vs. 56.94 ± 9.5 years, *p* > 0.05). The differences between the two groups were the treatment type and the type of stent. More patients in the local heparinized group received coiling alone and more patients in the systemic heparinized group received stent-assisted embolization (56.3 vs. 66%, *P* = 0.009). Dense network stents were more commonly used in the local heparinization group than in the systemic heparinization group (57.1 vs. 28.6%, *P* = 0.012). Table 1 shows comparative data between the local heparinization group and systemic heparinization group.

**Table 1:** Demographic parameters and distribution of patients who received local and systemic heparinization treatment for aneurysms.

	**Local heparinization**	**Systemic heparinization**	***p-*value**
No. of patients	91 (64.5)	50 (35.5)	
Age (years), mean ± SD	59.74 ± 10.7	56.94 ± 9.5	0.125
Female sex	63 (69.2)	39 (78.0)	0.265
Arterial hypertension	49 (53.8)	31 (60.8)	0.350
Diabetes mellitus	4 (4.4)	4 (8.0)	0.454
Aneurysm size no. (mm)	96 (64.4)	53 (37.6)	0.322
<5	54 (56.3)	23 (43.4)	
5–10	38 (39.6)	27 (50.9)	
>10	4 (4.2)	3 (5.7)	
Aneurysm site			0.538
Anterior communicating artery aneurysm	30 (31.3)	11 (20.8)	
Middle cerebral aneurysm	15 (15.6)	11 (20.8)	
Internal carotid artery aneurysm	45 (46.9)	28 (52.8)	
Posterior circulation aneurysm	6 (6.3)	3 (5.7)	
**Treatment type**			
Coiling alone	54 (56.3)	18 (34.0)	0.009
Stent–assisted coiling	42 (43.8)	35 (66.0)	
**Type of stent**			
Laser engraving stent	18 (42.9)	25 (71.4)	0.012
Dense network stent	24 (57.1)	10 (28.6)	

In the local heparinization group, 67 (69.8%) patients had abnormal DWI after the operation, including 37 (38.5%) patients in the coil embolization group and 30 (31.3%) patients in the stent-assisted embolization group.

A total of 36 cases (67.9%) with abnormal DWI after the operation were found in the systemic heparinization group, including 10 cases (18.9%) in the coil embolization group and 26 cases (49.1%) in the stent-assisted embolization group. There were no statistically significant differences in the incidence of DWI abnormalities between the local heparinization group and systemic heparinization group, including the subgroups (Table 2).

**Table 2:** Grouping of the heparinization schemes and analysis of cerebral ischemia.

**Heparinization schemes**	**Abnormalit, No. (%)**	**No DWI abnormality, No. (%)**	***P–*value**
Systemic heparinization	36 (67.9)	17 (32.1)	0.813
Local heparinization	67 (69.8)	29 (30.2)	
Systemic heparinization Coil embolization	10 (18.9)	8 (15.1)	0.551
Stent–assisted coiling	26 (49.1)	9 (17.0)	
Local heparinization Coil embolization	37 (38.5)	17 (17.7)	
Stent–assisted coiling	30 (31.3)	12 (12.5)	

**Figure 1:**
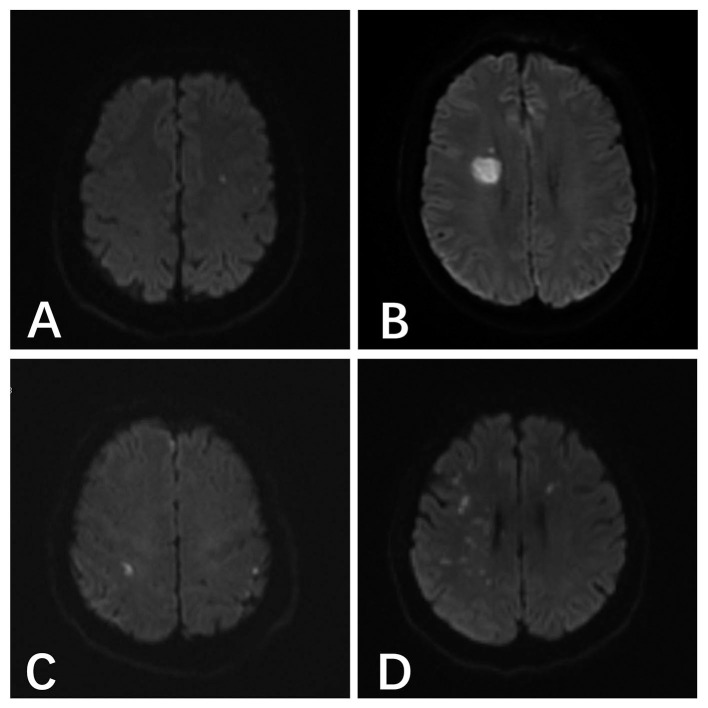
**(A)** A 46-year-old male patient with anterior communicating aneurysm underwent pure spring coil embolization under systemic heparinization, and the postoperative result indicated the acute cerebral infarction focus in the left parietal temporal lobe. **(B)** A 48-year-old male patient with right posterior communicating aneurysm underwent stent-assisted embolization under local heparinization, and the postoperative result indicated the acute cerebral infarction in the right lateral ventricle. **(C)** A 54-year-old male patient with right posterior communicating aneurysm underwent pure spring coil embolization under local heparinization, and the postoperative result indicated the abnormal DWI signals in the bilateral parietal lobes. **(D)** A 55-year-old female patient with a right middle cerebral aneurysm underwent stent-assisted embolization under systemic heparinization, the postoperative result indicated the acute cerebral infarction in the right parietal temporal lobe and the left frontal lobe.

The most common type of DWI abnormality in the systemic heparinization group was A (50%, 18/36). The most common type of DWI abnormality in the local heparinization group was also A (32.8%, 22/67). In the subgroups receiving coiling alone and stent-assisted coiling, the most common type of DWI abnormality in the systemic heparinization group was A, and the most common type of DWI abnormality in the coiling alone group with local heparinization was A (40.5%, 15/37). But, the most common DWI abnormality in the stent-assisted coiling group with local heparinization was C (36.7%, 11/40). The distribution of DWI abnormalities in the different groups showed no significant difference [(*P* > 0.05) (Table 3, Figure 1)].

**Table 3:** Distribution results of the heparinization method and DWI abnormality.

**Heparinization way**	**A, No. (%)**	**B, No. (%)**	**C, No. (%)**	**D, No. (%)**	**P–value**
Systemic heparinization	18 (50.0)	6 (16.7)	6 (16.7)	6 (16.7)	0.307
Local heparinization	22 (32.8)	11 (16.4)	20 (29.9)	14 (20.9)	
Systemic heparinization Coil embolization	4 (40.0)	3 (30.0)	2 (20.0)	1 (10.0)	0.520
Stent–assisted embolization	14 (53.8)	3 (11.5)	4 (15.4)	5 (19.2)	
Local heparinization Coil embolization	15 (40.5)	6 (16.2)	9 (24.3)	7 (18.9)	
Stent–assisted embolization	7 (23.3)	5 (16.7)	11 (36.7)	7 (23.3)	

The univariate analysis results showed that there were no significant differences among age, sex, history of arterial hypertension, diabetes, hyperlipidemia, location and size of aneurysm, ruptured aneurysm, heparinization scheme, intraoperative hypotension, operation time ≥ 120 min, embolization mode of the aneurysm and postoperative DWI abnormality (*P* > 0.05). Compared with the dense network stent, the laser engraving stent was more prone to DWI abnormalities (66.1 vs. 33.9%, *P* = 0.003) (Table 4).

**Table 4:** Univariate analysis results of DWI abnormalities after endovascular treatment for intracranial aneurysms.

**Clinical factors**		**DWI abnormality, No. (%)**	**No DWI abnormality, No. (%)**	***P*–value**
Age (year)	≤ 60	54 (55.7)	27 (61.4)	0.526
	>60	43 (44.3)	17 (38.6)	
Sex	Male	26 (26.8)	13 (29.5)	0.736
	Female	71 (73.2)	31 (70.5)	
Diabetes	Yes	7 (7.2)	1 (2.3)	0.435
	No	90 (92.8)	42 (97.7)	
History of stroke	Yes	9 (9.3)	2 (4.5)	0.503
	No	88 (90.7)	42 (95.5)	
Hypertension	Yes	58 (59.8)	22 (50.0)	0.277
	No	39 (40.2)	22 (50.0)	
Hyperlipidemia	Yes	68 (70.1)	26 (65.0)	0.558
	No	29 (29.9)	14 (35.0)	
Ruptured	Yes	67 (65.0)	29 (63.0)	0.813
	No	36 (35.0)	17 (37.0)	
Aneurysm is on the left	Yes	55 (53.4)	26 (56.5)	0.724
	No	48 (46.6)	20 (43.5)	
Aneurysm location	Anterior communicating artery aneurysm	25 (24.3)	16 (34.8)	0.324
	Middle cerebral aneurysm	20 (19.4)	6 (13.0)	
	Internal carotid artery aneurysm	50 (48.5)	23 (50.0)	
	Posterior circulation aneurysm	8 (7.8)	1 (2.2)	
Aneurysm size (mm)	<5	57 (55.3)	20 (43.5)	0.399
	5–10	41 (39.8)	24 (52.2)	
	>10	5 (4.9)	2 (4.3)	
Wide neck aneurysm	Yes	28 (27.2)	9 (19.6)	0.320
	No	75 (72.8)	37 (80.4)	
Heparinization mode	Local heparinization	67 (65.0)	29 (63.0)	0.813
	Systemic heparinization	36 (35.0)	17 (37.0)	
Intraoperative hypotension	Yes	37 (36.6)	17 (37.0)	0.970
	No	64 (63.4)	29 (63.0)	
Operation time ≥120min	Yes	61 (59.2)	23 (50.0)	0.294
	No	42 (40.8)	23 (50.0)	
Stent used	Yes	56 (54.4)	21 (45.7)	0.325
	No	47 (45.6)	25 (54.3)	
Type of stent	Laser engraving stent	37 (66.1)	6 (28.6)	0.003
	Dense network stent	19 (33.9)	15 (71.4)	

Variables including female gender, age >60 years old, aneurysm on the left side, location and size of aneurysm, local heparinization, intraoperative hypotension, operation time ≥120 min and stent usage were included in the multivariate logistic regression analysis as independent variables. (Appendix Table A1). The results showed that there was no significant correlation among female gender, age >60 years old, aneurysm on the left side, location and size of aneurysm, local heparinization, intraoperative hypotension, operation time ≥120 min and DWI abnormalities after endovascular treatment for intracranial aneurysms. There was a significant correlation between the engraving stent (OR 4.711, 95% CI 1.512–14.584, *P* = 0.007) and DWI abnormalities after endovascular treatment for intracranial aneurysms (Table 5).

**Table 5:** Multivariate analysis results of clinical data.

**Clinical data**		**OR (95% CI)**	***P*–value**
Sex (female)		0.822 (0.317–2.131)	0.687
Age (>60 years)		1.395 (0.603–3.224)	0.436
Left side		0.879 (0.391–1.974)	0.754
Location of aneurysm	Anterior communicating artery aneurysm	0.167 (0.017–1.688)	0.129
	Middle cerebral aneurysm	0.530 (0.049–5.780)	0.603
	Internal carotid artery aneurysm	0.254 (0.027–2.399)	0.231
Size of aneurysm (mm)	<5	0.931 (0.126–6.853)	0.944
	5–10	0.502 (0.066–3.791)	0.504
Local heparinization		1.742 (0.725– 4.183)	0.214
Intraoperative hypotension		0.880 (0.373–2.078)	0.770
Operation time ≥120min		0.988 (0.427–2.290)	0.978
Dense network stent		0.699 (0.282– 1.734)	0.440
Laser engraving stent		4.711 (1.521–14.584)	0.007*

* *P < 0.05*.

Among 103 cases of abnormal DWI lesions, 91 patients (88.3%) had no symptoms and 12 patients (11.7%) had symptoms, including five patients with headache, three patients with vomiting, two patients with allergies, one patient with somnolence, and one patient with chest distress. All patients recovered well without a secondary hemorrhage or neurological sequelae when discharged from the hospital.

## Discussion

Intracranial aneurysm is a highly dangerous hemorrhagic cerebrovascular disease. Rebleeding in patients with ruptured intracranial aneurysms may occur at any time during the acute phase, thus requiring a more timely operation to achieve the purpose of treatment, especially for patients with a large amount of blood loss, who may need other invasive operations immediately after embolization. The existence of these factors challenges the safety and necessity of traditional systemic heparinization. In this study, 91 patients with 96 acute hemorrhagic aneurysms were treated by endovascular embolization with local heparinization, including 67 cases (69.8%) who showed DWI abnormalities. These findings are consistent with the incidence rate of visible ischemic lesions on DWI after postoperative intravascular treatment reported by previous studies, which is between 10 and 77% (2–5). Thus, it can be seen that the application of local heparinization does not increase the risk of intraoperative cerebral ischemic events. In terms of postoperative DWI abnormalities, there was no significant difference (69.8 vs. 67.9%, *P* = 0.813) in its incidence in the local heparinization group and systemic heparinization group. In the subgroup analysis of coiling alone and stent-assisted coiling, there were no significant differences in DWI abnormalities. At the same time, there were no bleeding events in the two groups during hospitalization, which indicated that the application of local heparinization in the endovascular treatment of intracranial aneurysms did not increase the risk of bleeding and ischemic complications compared with systematic heparinization.

The anticoagulant effect of heparin is mediated largely through its interaction with antithrombin III(ATIII).this produces a conformational change in ATIII and so markedly accelerates its ability to inactivate the coagulation enzymes thrombin (factor IIa), factor Xa, and factor IXa. Heparin is probably the most commonly used intraoperative drug that is considered to reduce the risk of intraoperative thromboembolic events (9–11). Most centers apply systemic heparinization in the endovascular treatment of intracranial aneurysms. Usually, the heparin dosage for vein mass injection at the beginning of the operation ranges from 3,000 to 5,000 U, followed by a continuous intravenous drip at a rate of 20–40 U/kg/h, and the activated clotting time (ACT) is maintained between 200 and 300 s to avoid the complications of thromboembolism (9, 12–14). The World Federation of Interventional Neuroradiology (WFITN) recommends 5,000 U of heparin for intravenous infusion, followed by a 1,000 U/h continuous intravenous drip, while maintaining an ACT value of 200 s (13). We studied the replacement of systemic heparinization with local heparinization in the treatment of ruptured aneurysm. The catheter flashing solution contained 2,000 U of heparin per 500 ml. Through the analysis of the distribution of different heparinization schemes and cerebral ischemia events on DWI, we found that the most common type of ischemic event distribution was A (*n* = 40, 38.8%) in this series. Meanwhile, the comparison of ischemic event distribution types between the systemic heparinization group and the local heparinization group did not show significant differences, which indicated that different schemes of heparinization did not affect the distribution of abnormal ischemic events on DWI, and there was no difference between the different methods of heparinization and DWI abnormalities. At the same time, the distribution of aneurysms at different locations showed no difference in this study (*P* = 0.298). We considered that these DWI abnormalities may be related to the operation of the patient, and these lesions are correlated with the specific locations of the aneurysm according to reports by some researchers (15, 16).

The incidence of thromboembolic events after cerebral aneurysm coil embolization was still high, and previous studies have shown that various factors are positively correlated with postoperative ischemic events, including older age, dyslipidemia, diabetes, history of ischemic stroke, leukodystrophy, rupture of aneurysms, multiple aneurysms, larger neck size and longer operation time (5, 15–18). Stent-assisted embolization was also considered to be the cause of the significant increase in the risk of embolism (19). In our study, the incidence of DWI abnormalities of the two types of stents after the operation showed statistical significance (laser engraving stent, *n*=37, 66.1%; dense network stent, *n* = 19, 33.9%; *P* = 0.007). In the subsequent multivariate analysis, it was indicated that engraving stents were also correlated with asymptomatic ischemic events (*P* < 0.05), which may be related to the stent parameters, stent implantation operation method, whether the stent was well-attached during the operation, and the influence of stent implantation on hemodynamics. These factors may influence the conversion of intracranial aneurysms.

Previous studies have found that patients with an intracranial aneurysm on the left side had a higher incidence of postoperative cerebral ischemia. The results of this study showed that the aneurysm located on the left side (*n* = 55, 53.4%) showed abnormal DWI after operation, but there was no correlation on the left side in univariate analysis. In a prospective study of 39 patients with unruptured aneurysms, Sim et al. (16) proposed that the left admission passage may increase the possibility of asymptomatic multiple sporadic microemboli after the aneurysm embolization operation. It was suggested that the left great vessels, especially the left common carotid artery, tended to be more difficult to perform surgery on due to their larger angle with the aortic arch, so left-sided aneurysms undergoing surgery were more prone to these ischemic complications. At the same time, some authors have speculated that these multiple microemboli may originate from the intraoperative removal of small blood clots, the atherosclerotic plaque from the aortic arch or the great vessel wall via catheter operation (20). However, there was no significant difference in the location of DWI abnormalities in our study.

The operation time as a risk factor for postoperative cerebral ischemia has also been documented in previous studies. The incidence of cerebral ischemia lesions after embolization of the aneurysm increased with the increase of the operation time (17). In this study, we took an operation time ≥120 min as a marker of a long operation. Correlation analysis between a single factor and multiple factors with DWI abnormalities was conducted, and none of the results showed statistical significance. Park et al. (21) verified that the occurrence of microemboli was positively correlated with the operation time in univariate analysis; however, such a relation was not confirmed in subsequent multivariate analysis. A longer duration of the operation may mean that the aneurysm grows in difficult locations and the operator encounters increased difficulties during the operation. Lee et al. (17) suggested that shortening of the operation time is the most effective way that surgeons can reduce the incidence of thromboembolic events after embolization. Therefore, before the operation, the operator should work out a detailed operation plan according to the patient's condition and aneurysm condition to ensure the operation goes smoothly and to reduce intraoperative and postoperative complications of the patient. At the same time, we also assessed the relation between intraoperative hypotension and the occurrence of DWI abnormalities for the first time. Although intraoperative hypotension means that the patient has lower cerebral perfusion and a greater risk of embolization events, a correlation between intraoperative hypotension and DWI abnormalities was not shown in our results.

No significant correlation was found between ruptured aneurysms and postoperative DWI abnormalities (*P* > 0.05) in our study. In the treatment of ruptured aneurysms, some studies have shown that thrombolytics, such as tirofiban, can effectively dissolve the thrombus or embolus during the operation (22). Tirofiban is a non-peptide antagonist of platelet glycoprotein (GP) IIb/IIIa receptor, which can inhibit platelet aggregation, and the patient's normal platelet function is restored within 4 h after drug withdrawal (23). We used tirofiban to prevent thromboembolic events during stent-assisted embolization with local heparinization. Although the incidence of DWI abnormalities with local heparinization was 69.8%, no further rupture or bleeding occurred after the operation, which indicated that the application of tirofiban was safe with local heparinization, even though long-term injection of tirofiban may have a risk of cerebral hemorrhage.

How to reduce ischemic complications in clinical applications has always puzzled clinicians. One strategy is to use antiplatelet drugs prior to the selective aneurysm coil embolization operation, as it has been proven to reduce the risk of clinical thromboembolic events (24, 25). How to reduce the occurrence of microemboli events with abnormal DWI during the aneurysm operation has also been studied by many researchers. Kim et al. (26) obtained the incidence of diffusion-weighted imaging-high signal intensity (DWI-HIS) lesions in a conventional group (89.2%, 33/37) and an improved group (26.5%, 9/34) in a study of 71 patients with unruptured cerebral aneurysms by deliberately aspirating the contents of the microcatheter after releasing each spring coil, and the difference was statistically significant (*P* < 0.0001). Lim et al. (27) showed that prior use of a heparin bolus dosage above 2,000 U during cerebral aneurysm embolization may reduce cerebral ischemic events after embolization. The use of new-generation blood flow guides can also reduce the incidence of such complications. Pikis et al. (28) found that new-generation blood flow guides significantly reduced the incidence of such complications in a study of 33 patients with unruptured aneurysms, with a DWI positive lesion rate of 16.66%, which also requires further study.

### Limitations

Our study has several limitations. First, this is our single center's exploration of different heparinization schemes. Second, we cannot exclude the effect of ruptured aneurysms on vascular stimulation and ischemia in this study. Third, since we did not conduct preoperative MRI examinations, some ischemic lesions observed on DWI after the operation may have occurred before the treatment of intracranial aneurysms.

## Conclusion

Our study shows that local heparinization is safe and effective in endovascular embolization for intracranial aneurysms and does not increase the risks of cerebral ischemia and bleeding complications compared with traditional systemic heparinization. DWI abnormalities after intracranial aneurysm embolization are a serious problem, and how to reduce the occurrence of DWI abnormalities may require more clinical data experiments and basic experimental studies.

## Data Availability Statement

The original contributions presented in the study are included in the article/supplementary materials, further inquiries can be directed to the corresponding author/s.

## Author Contributions

LZ contributed to the preparation of the manuscript and data collection. XZ contributed to revising the manuscript. YL and CD contributed to data analysis and interpretation. YW and HY contributed to the experi-mental design and manuscript revision. All authors contributed to the article and approved the submitted version.

## Conflict of Interest

The authors declare that the research was conducted in the absence of any commercial or financial relationships that could be construed as a potential conflict of interest. The handling editor declared a shared affiliation, with several of the authors YW, HY at time of review.

## Appendix

**Table A1:** Assignment table of single-factor and multi-factor logistics regression analysis for DWI abnormalities after endovascular treatment for intracranial aneurysms.

**Item**	**Assignment**
DWI abnormality	Yes = 1, No = 0
Sex	Female = 1, Male = 2
Age	Above 60 years = 1, Below 60 years = 2
Aneurysm is on the left	Left side = 1, Right side = 2
Ruptured	Yes = 1, No = 2
Aneurysm location	Anterior communicating artery aneurysm = 1, Middle cerebral aneurysm = 2, Internal carotid artery aneurysm = 3, post-circulation aneurysm = 4 (dummy variable)
Aneurysm size	<5 = 1, 5–10 = 2, Above 10 = 3 (dummy variable)
Heparinization mode	Local heparinization = 1, Systemic heparinization = 2
Intraoperative blood pressure condition	Hypotension occurred during operation = 1, Hypotension did not occur during operation = 2
Operation duration	Operation time ≥120 min = 1, Operation time <120 min = 2
Stent usage condition	Dense network stent = 1, Laser engraving stent = 2, Unused stent = 3 (dummy variable)
